# Self-assembly of sodium caprate in simulated intestinal fluids and with a therapeutic peptide: A small-angle neutron scattering study

**DOI:** 10.1016/j.ijpx.2026.100641

**Published:** 2026-08-16

**Authors:** Shahina Akter, L. Magnus Bergström, Per Hansson, Joachim Kohlbrecher, James Doutch, Shakhawath Hossain, Per Larsson

**Affiliations:** a Department of Pharmacy, Uppsala University, 751 23 Uppsala, Sweden; b Department of Medicinal Chemistry, Uppsala University, Box 574, SE-751 23 Uppsala, Sweden; c PSI Center for Neutron and Muon Sciences, 5232 Villigen PSI, Switzerland; d ISIS Neutron and Muon Source, STFC, Rutherford Appleton Laboratory, Harwell Campus, Didcot OX11 0QX, Oxon, United Kingdom

## Abstract

The self-assembly behavior of sodium caprate (C10), a widely used intestinal permeation enhancer, was characterized under intestinally relevant conditions using small-angle neutron scattering (SANS) with contrast variation. Systems containing 100 mM C10, alone and in fasted-state (FaSSIF) and fed-state (FeSSIF) simulated intestinal fluid, together with 300 mM C10 in the presence of the therapeutic peptide octreotide, were investigated at pH 6.5 and 8.5. At pH 6.5, C10 alone formed coexisting ellipsoidal aggregates, vesicles, and large droplets. Addition of FaSSIF promoted co-assembled mixed structures, including large ellipsoidal aggregates, vesicles, and bilayer-like morphologies, while FeSSIF shifted the system further toward bilayer discs. The most pronounced structural reorganization occurred in the presence of octreotide, where C10 aggregates transformed into large bilayer discs with aggregation numbers approaching 18,000. At pH 8.5, all systems converge to small spherical micelles (radius 15–20 Å), with the notable exception of the C10-octreotide system, which forms prolate rod-like micelles. Contrast-dependent fitting showed that octreotide promotes axial micellar elongation without substantially altering radial packing, indicating amphiphilic cosurfactant-like behavior rather than peptide incorporation into the hydrophobic core. Coarse-grained molecular dynamics simulations supported this interpretation, showing rod-like aggregate formation in the presence of octreotide and cosurfactant-like behavior, with hydrophobic residues inserted into the micelle and hydrophilic Lys and Thr residues positioned at the interface. These findings demonstrate that intestinal fluid composition and peptide-excipient interactions are principal determinants of C10 aggregate architecture, providing a foundation for the rational design of caprate- and fatty-acid-based absorption enhancer systems for oral peptide delivery.

Author keywords sodium caprate, small-angle neutron scattering, contrast variation, intestinal fluid, octreotide, cosurfactant, permeation enhancer.

## Graphical abstract

**Figure:**
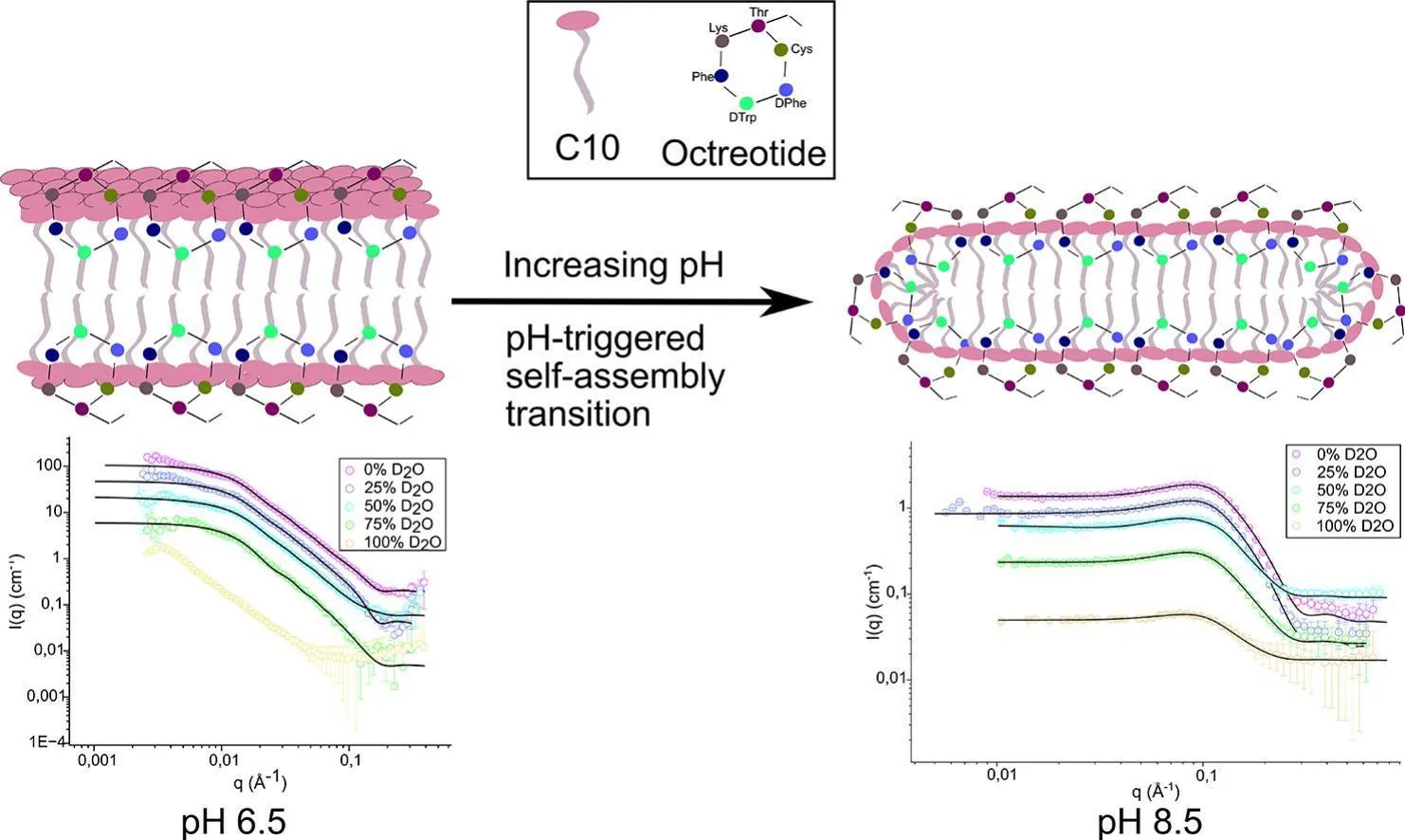

## 1 Introduction

Amphiphilic surfactants self-assemble into supramolecular structures such as micelles, vesicles, and mixed aggregates in aqueous environments (Nagarajan, 2011), making them valuable components in drug delivery systems owing to their ability to solubilize poorly water-soluble drugs, modulate release profiles, and enhance drug permeability across biological membranes (Maher et al., 2023). Among anionic surfactants used in pharmaceutical applications, medium-chain fatty acids (MCFAs) and their salts have attracted particular attention (Maher et al., 2009). Sodium caprate (C10), sodium caprylate (C8), and the structurally distinct permeation enhancer salcaprozate sodium (SNAC) have been developed over more than two decades as components of proprietary drug delivery platforms, particularly for enhancing the oral bioavailability of peptide-based therapeutics (Maher et al., 2009; Hossain et al., 2019; Twarog et al., 2019; Buckley et al., 2018). In aqueous solutions, fatty acids self-assemble into aggregates of varying size and structure depending on concentration (Namani and Walde, 2005; Kanicky and Shah, 2003), which can influence their effectiveness as permeability enhancers by regulating the availability of free monomers at the intestinal absorption site (Maher et al., 2009). Among the MCFA permeation enhancers, C12 and C14 possess low critical micelle concentrations (CMC) (Hossain et al., 2019; Prisle et al., 2008), limiting the availability of free monomers for membrane interaction (Kneiszl et al., 2022), whereas C8, despite its high CMC, interacts less efficiently with lipid bilayers as its shorter hydrocarbon chain does not penetrate sufficiently into the hydrophobic core of the membrane to cause significant disruption (Kneiszl et al., 2022). C10 exhibits a moderate CMC and a comparatively greater ability to disrupt membrane integrity (Kneiszl et al., 2022), making it particularly suitable as a permeation enhancer (Heerklotz, 2008). Its clinical performance, however, is variable and often falls short of alternative enhancers such as salcaprozate sodium (SNAC); recent in vivo comparisons in rats showed markedly lower octreotide bioavailability when co-administered with C10 than with SNAC (Fredholt et al., 2026).

The self-association of C10 in aqueous solution alters its acid-base behavior, causing the apparent pKa to shift from its intrinsic value of approximately 4.8 to around 7, depending on concentration (Namani and Walde, 2005; Kanicky and Shah, 2003). The aggregation behavior is highly sensitive to the relative proportions of its ionized and unionized species (Namani and Walde, 2005; Morigaki et al., 2003). At alkaline pH (> 8), where the ionized species predominate, C10 assembles into micelles; when the pH decreases and the proportion of the unionized form increases, larger aggregates form. The CMC is comparatively high (50–100 mM) under alkaline conditions, as the highly charged headgroups generate strong electrostatic repulsion between surfactant molecules, disfavoring micelle formation (Namani and Walde, 2005). At lower pH levels (6.5–7.5), when C10 is present predominantly in its unionized form, the CMC drops to around 10–20 mM due to the corresponding reduction in surface charge density (Shirahama and Kashiwabara, 1971). The CMC also depends on the type of counterions and the ionic strength of the solution, further complicating aggregation behavior (Namani and Walde, 2005). The self-aggregation of C10 surfactants and medium-chain fatty acids has been characterized in previous research. The primary focus of these studies was on critical micelle concentrations, micellization energetics, and the effects of molecular structure, concentration, pH, temperature, ionic strength, and salt composition in relatively simple aqueous or buffered conditions (Hossain et al., 2019; Akter et al., 2026; Chakraborty and Moulik, 2007; Rodríguez-Pulido et al., 2010). However, the size and morphology of the assemblies it forms under physiologically relevant conditions, particularly because the complex composition of intestinal fluids (Clulow et al., 2017) and the presence of therapeutic peptides, remain poorly understood. Understanding these structural features is essential, as they can strongly influence stability, drug loading, and transport behavior. While established nanocarriers such as lipodiscs and liposomes are often preferred for drug delivery because of their proven biocompatibility (Karmaker et al., 2025), a detailed understanding of the self-assembled structures formed by C10 is needed to assess its potential as a formulation platform for oral peptide delivery.

The aggregation behavior of C10 may be best characterized for example with small-angle neutron scattering (SANS). This has the advantage that it directly probes nanoscale structures in solution and gives information on the size, shape, internal organization, and interparticle interactions of the aggregates as an ensemble average (Matsuoka, 1996). SANS has been widely applied to surfactant self-assembly (Hayter and Penfold, 1983), to the characterization of bile salt-phospholipid mixed micelles under simulated intestinal conditions (Rezhdo et al., 2017), and to drug-surfactant systems of pharmaceutical relevance (Clulow et al., 2017). Importantly, changing the contrast by selective deuteration or changing the H₂O/D₂O ratio can tell the difference here between C10-rich assemblies, peptides, and other parts of the media (Heller, 2010; Krueger, 2022). The contrast-variation approach is especially powerful for multi-component systems, since selective visibility of individual components allows the internal composition and spatial distribution of species within aggregates to be determined.

CG-MD complements experimental approaches by offering molecular-level insight into aggregation formation, size, shape, composition and dynamics (Hossain et al., 2019; Akter et al., 2026; Hossain et al., 2020). CG-MD studies of medium-chain fatty acid aggregation have provided quantitative insight into the free monomer concentration, aggregation kinetics, and pH-dependent morphology transitions (Hossain et al., 2019). It can also demonstrate the impact of individual factors, including pH, intestinal-fluid components, and peptide interactions, on aggregation in physiologically relevant conditions. Used together, SANS and CG-MD provide a structural picture of C10 aggregates in physiologically relevant environments that goes beyond what either method could offer alone.

In this study, we examine the aggregation morphology and interaction behavior of C10 under physiologically relevant conditions at two different pH levels. The self-assembly of C10 was investigated in simulated intestinal fluid under both fasted (FaSSIF) and fed (FeSSIF) states, as well as in the presence of the therapeutic peptide octreotide, using small-angle neutron scattering (SANS) and light scattering techniques. Octreotide was selected as a model therapeutic peptide based on previous reports demonstrating enhanced bioavailability when co-administered with C10 (Berg et al., 2021). Building on these findings, the present study aimed to elucidate the mode of interaction between octreotide and C10 within self-assembled aggregates, specifically evaluating whether the peptide acts as an amphiphilic cosurfactant, is incorporated into the micellar core, or preferentially localizes at the aggregate interface. Distinguishing between these interaction modes is important because each has different implications for drug loading capacity, aggregate stability, and the availability of free monomers for membrane permeation enhancement. Each of the three interaction modes produces a distinct signature in the SANS contrast-variation profile, particularly in how the aggregate cross-sectional dimensions respond to changes in solvent contrast, and can be further tested against CG-MD predictions of molecular-scale organization. Discriminating between them therefore provides a structural foundation for interpreting the in vivo behavior of C10-octreotide formulations and, more broadly, for designing peptide-enhancer combinations rationally.

## 2 Experimental

### 2.1 Materials

Sodium decanoate (sodium caprate, C10; purity ≥98%), monobasic potassium phosphate, dibasic potassium phosphate, and deuterium oxide were purchased from Sigma-Aldrich. Deuterated sodium decanoate-d₁₉, d-C10 was obtained from Qmx Laboratories (UK) (purity ≥98.9%) and from the Deuteration & Macromolecular Crystallization (DEMAX) facility at the European Spallation Source ERIC (purity >98.3%). 3F powder (FaSSIF and FeSSIF v2) was sourced from Biorelevant (UK). Octreotide acetate was purchased from Chemos (Altdorf, Germany).

### 2.2 Sample preparation

**Fig. 1:**
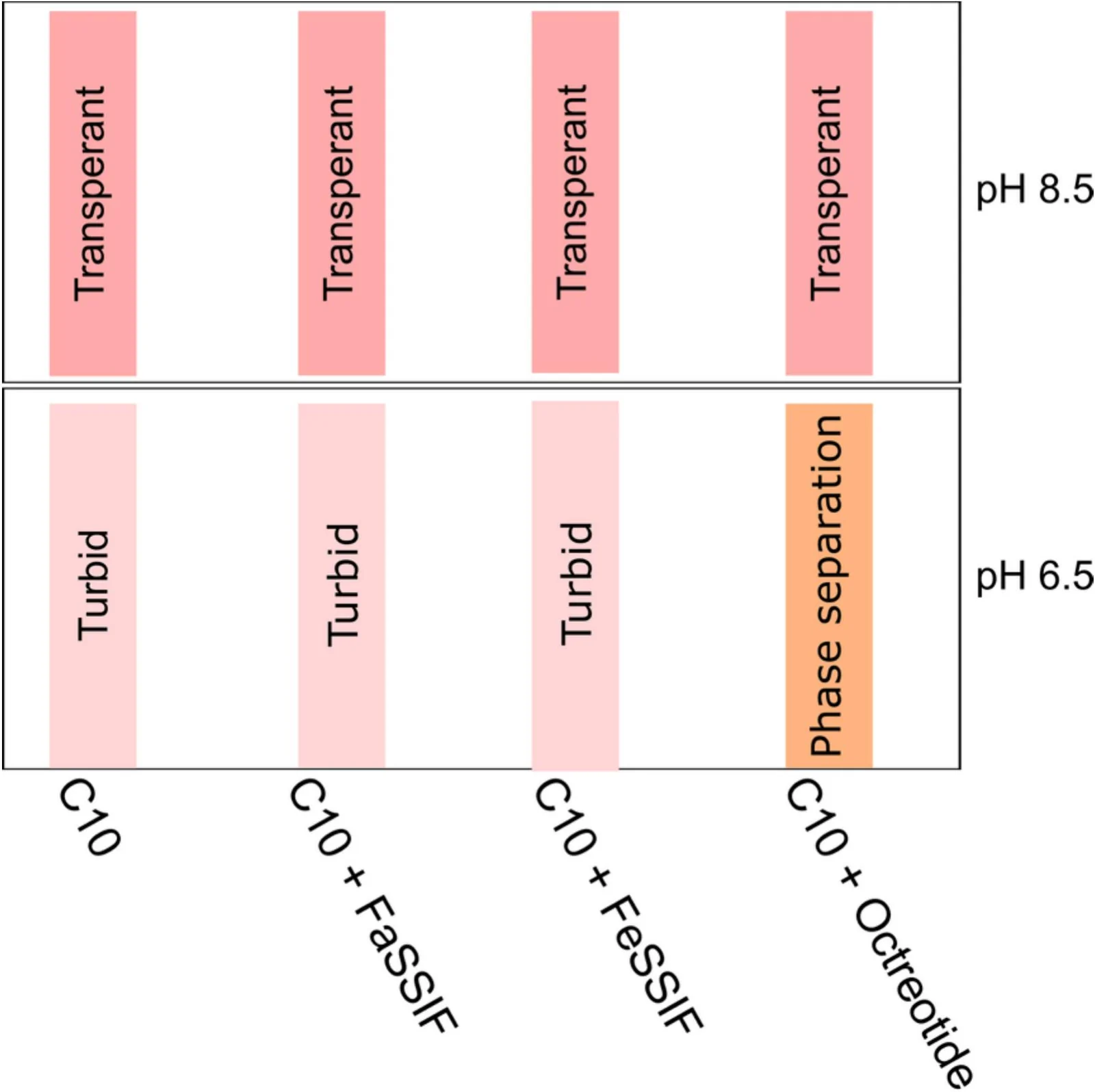
Visual evaluation of C10 aqueous solutions in the presence of different components at pH 6.5 and pH 8.5. Samples containing C10 alone and C10 in combination with FaSSIF, FeSSIF, or octreotide were visually examined and classified according to their appearance as transparent, turbid, or phase-separated. At pH 6.5, C10 alone and mixtures containing FaSSIF or FeSSIF appeared turbid, whereas the mixture of C10 with octreotide showed phase separation. In contrast, all samples were transparent at pH 8.5.

Phosphate buffers (0.1 M) in H₂O (h-PB) and D₂O (d-PB) were prepared using monobasic and dibasic potassium phosphate, following the procedure outlined by Conrad, M. L., et al. (2009) (Conrad et al., 2009). The buffers were left to equilibrate for at least 24 h before measuring pH and pD. The difference between pH and pD was measured directly and found to be 0.1 units. All samples listed in Table 1 were prepared using 0.1 M h-PB and d-PB buffers. The desired pH or pD was adjusted using 0.1 M HCl/DCl and 0.1 M NaOH. Before the experiment, all samples were allowed to equilibrate at room temperature. We investigated C10/d-C10 at a physiologically relevant concentration of 100 mM, both alone and in the presence of fasted-state and fed-state simulated intestinal fluids (FaSSIF and FeSSIF, respectively). FaSSIF and FeSSIF media were prepared from the same commercial SIF powder (3F powder). The FaSSIF medium contained sodium taurocholate (3 mM) and phospholipids (0.75 mM), corresponding to a total powder concentration of 2.2 mg/mL. The FeSSIF medium contained sodium taurocholate (15 mM) and phospholipids (3.75 mM), corresponding to a total powder concentration of 11.2 mg/mL. Thus, FaSSIF and FeSSIF differed only in the concentration of the same SIF powder used for preparation. Peptide-free systems were examined at 100 mM C10 to analyze C10 aggregation in the chosen intestinal environments. A distinct formulation-related system including 300 mM d-C10 and 2 mM octreotide was analyzed according to settings previously reported by Berg et al. to improve intestinal absorption (Berg et al., 2022). Different solvent ratios were used in the SANS experiments depending on the total beamtime available. (See Fig. 1.)

**Table 1:** Summary of systems studied using Small-Angle Neutron Scattering (SANS).

Systems	Solvent ratio h-PB/d-PB	Conc. of C10 [mM]	Instrument
C10 d-C10 d-C10 with FaSSIF^a^	0, 20, 40, 60, 80 and 100%	100	SANS-I, PSI
d-C10 with FeSSIF^b^ d-C10 with octreotide	0, 25, 50, 75, and 100%^c^	50, 100 300	ZOOM, ISIS

a : FaSSIF is mainly bile salts (Na-taurocholate, 3 mM) and phospholipids (0.75 mM). The concentration of FaSSIF was 2.2 mg/mL.

b : FeSSIF is mainly bile salts (Na-taurocholate, 15 mM) and phospholipids (3.75 mM). The concentration of FeSSIF was 11.2 mg/mL.

c : Number of solvent contrasts was reduced because the limitation of the beamtime.

### 2.3 Small-angle neutron scattering (SANS)

SANS experiments were conducted using the SANS-I instrument at the PSI Center for Neutron and Muon Sciences in Villigen, Switzerland (Wagner, 2000; Instrument, S.-I.S.A.N.S, n.d.). The samples were contained in 2 mm Hellma quartz cells during the experiment. The measurements were performed at room temperature (19 °C) with a neutron wavelength of λ = 6 Å and a wavelength resolution of Δλ/λ = 10% (Wagner, 2000). To achieve a comprehensive wave vector transfer (q) range from 0.00327 to 0.341 Å^−1^, data were collected at three sample-to-detector distances: 2 m, 4 m, and 8 m. The raw data were processed using the BerSANS software (Keiderling, U. and G.S, 2002), which included radial averaging and corrections for both the electronic background and the empty cell background. For the detector calibration, a sample containing H₂O was measured to ensure homogeneous illumination of the detector, taking advantage of water's strong isotropic incoherent scattering. All data were converted to an absolute scale using H₂O as a reference.

Additional SANS experiments were performed on the ZOOM instrument at the ISIS neutron and muon spallation source at Rutherford Appleton Laboratory in Didcot, UK (*ZOOM diffractometer at the ISSI neutron source*, n.d.). The measurements were carried out at room temperature (20 °C) at sample-to-detector distances of 8 m and 4 m, corresponding to q-ranges of 0.002 to 0.387 Å^−1^ and 0.004 to 0.775 Å^−1^, respectively. 2 mm Hellma quartz cells were used as the sample container. Data reduction was conducted using the Mantid software (Keiderling, U. and G.S, 2002), which included the correction of sample transmission, detector efficacy, and scattering from an empty sample cell. The final data sets provided absolute scattered intensities, I (q) in cm^−1^, plotted against momentum transfer, q in Å^−1^.

### 2.4 Static light scattering

Static light scattering (SLS) experiments were conducted using an ALV/CGS-3/MD-4 multi-detection goniometer system equipped with ALV/LSE-5004 light-scattering electronics (ALV, Germany). The setup employed a 50 mW diode-pumped solid-state laser operating at a wavelength of 532 nm and fibre-optic single-detector units with a fibre-based beam splitter optimized for this wavelength.

### 2.5 Data analysis

The SANS data were analyzed using an in-house least-squares fitting program developed by Pedersen (Pedersen, 1997). Depending on the system under investigation, different fitting models (described in supplementary information) were employed, as summarized in Table 2. For the systems at pH 6.5, the data could be adequately described using form-factor models alone, without the inclusion of a structure factor; consequently, parameters related to interparticle interactions, such as surface charge density and aggregate volume fraction, could not be extracted. In contrast, some pH 8.5 systems—such as 300 mM C10 in the presence of octreotide—required the inclusion of both form and structure factors to achieve satisfactory fits. Detailed results from the SANS data analysis are summarized in Tables S1–S5 in the supplementary information. The SLS data did not display a clear Guinier regime and were therefore deemed unsuitable for reliable quantitative analysis. All the SLS data, together with the SANS data, are shown in the supplementary information.

**Table 2:** The models used to fit SANS data for C10 systems at pH 6.5 and pH 8.5 under different solution conditions. See model descriptions in the supplementary information.

Model Name	pH 6.5 systems	pH 8.5 systems
Ellipsoid	C10 (60–100% D_2_O) d-C10 (0–100% D_2_O)	–
Sphere	–	C10 (100% D_2_O) d-C10 (0% D_2_O) d-C10 with FaSSIF (0% D_2_O) d-C10 with FeSSIF (0–100% D_2_O)
Two ellipsoids	d-C10 with FaSSIF (0–40% D_2_O)	–
Bilayer discs modelled as flat cylinders	d-C10 with FaSSIF (60–100% D_2_O)	–
Bilayer coexistence with micelles	d-C10 with FeSSIF (0–100% D_2_O) d-C10 with octreotide (0–100% D_2_O)	–
Ellipsoid (with Structure factor)	–	d-C10 with octreotide (0–100% D_2_O)

### 2.6 Cryo-TEM

Three or four microliters of sample were applied to glow-discharged (20 mA, 0.39 mbar, 30 s, PELCO easiGlow™) holey carbon grids 2/2 Cu 300 mesh (Quantifoil Micro Tools GmbH). Grids were blotted at 20 °C and 100% humidity using filter paper (Whatman®) and plunge-frozen in liquid ethane using a Vitrobot Mark IV (Thermo Fisher Scientific). Vitrified grids were screened at the Cryo-EM Uppsala facility (Uppsala, n.d.) using 200 kV Glacios (Thermo Fisher Scientific) equipped with a Falcon 4i direct electron detector (Thermo Fisher Scientific) with Selectris Energy filter (Thermo Fisher Scientific) using 10 eV slit, at ×79,000 and ×49,000 nominal magnification.

### 2.7 Coarse-grained molecular dynamics (CG-MD) simulations

Coarse-grained molecular dynamics (CG-MD) simulations were performed using the Martini 3 force field (Souza et al., 2021) to investigate the 300 mM C10–2 mM octreotide system at pH 8.5. The C10 topology was adopted from our previous studies (Hossain et al., 2022), and the octreotide topology was generated using Martinize2 (Kroon et al., 2025) for Martini 3 coarse-grained proteins/peptides, applied to the all-atom structure utilized in our prior work (Hossain et al., 2023).

Simulations were performed using GROMACS 2025.1 (Abraham et al., 2015). The system was first energy-minimized using the steepest descent algorithm, followed by equilibration for 100,000 steps with a 10-fs time step. The Verlet cutoff scheme was used for both Coulombic interactions, treated with the reaction-field method, and van der Waals interactions, with a cutoff of 1.1 nm. Periodic boundary conditions were applied in all directions. Temperature was maintained at 303 K using the velocity-rescaling thermostat (Bussi et al., 2007) with a coupling constant of 1 ps. Pressure was applied isotropically at 1.0 bar using the Parrinello–Rahman barostat (Parrinello and Rahman, 1981) with a coupling constant of 12 ps and a compressibility of 3 × 10^−4^ bar^−1^ during the production simulation. Production simulations were then run with a 20-fs time step. To identify micelles, two molecules were considered to belong to the same micelle if any of their constituent beads were within a defined cutoff distance (0.6 nm) (Hossain et al., 2020).

## 3 Results and discussion

### 3.1 Sample description

Visual examination of C10 aqueous solutions in the presence of different components revealed three distinct states: transparent, turbid, and phase-separated. At pH 8.5, all samples—including C10 alone and C10 in combination with FaSSIF, FeSSIF, or octreotide—appeared transparent, indicating that C10 is essentially fully soluble under these conditions. At this pH, C10 exists predominantly above its critical micelle concentration (CMC), and the headgroups are highly ionized. As a result, C10 forms small, charged micelles with sizes on the order of a few nanometers, which contributes to the optical clarity of the solutions.

In contrast, at pH 6.5, C10 alone and C10 in combination with FaSSIF or FeSSIF produced turbid solutions. After several days of equilibration, the initially turbid C10 solution gradually separated into distinct phases. Furthermore, when C10 was mixed with the oppositely charged peptide octreotide, phase separation occurred within 10 min of preparation, resulting in distinct oily and aqueous phases. At lower pH, C10 is predominantly present in its unionized form, and the critical micelle concentration (CMC) increases. Under these conditions, C10 tends to form complex colloidal structures in solution. To characterize the size and morphology of these colloidal assemblies in C10 mixtures, SLS, SANS, Cryo-TEM, and CG-MD simulations were employed.

### 3.2 C10 and d-C10 in buffered solution

**Fig. 2:**
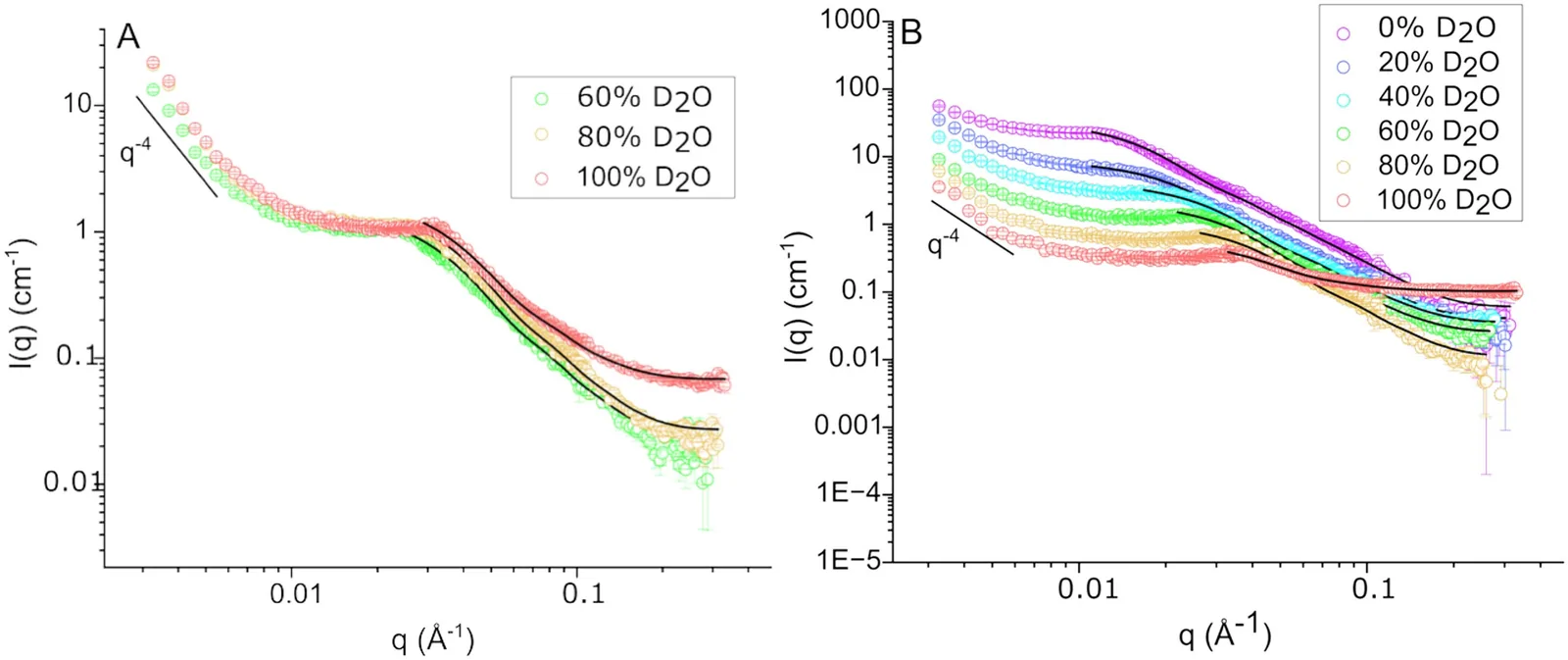
Small-angle neutron scattering (SANS) profiles and corresponding model fits for systems containing 100 mM C10 (A) and d-C10 (B) at pH 6.5. The model fit in the high-q region is consistent with the ellipsoidal model, and the slope q-4 in the low-q region indicates large particles.

Scattering intensity profiles for 100 mM C10 and d-C10 at pH 6.5 for various solvent ratios are shown in Fig. 2.

For the C10 system, scattering profiles suitable for model fitting were obtained at 60%, 80%, and 100% D_2_O contrasts (Fig. 2A), indicating the coexistence of small micelles and a dispersed phase of larger aggregates. The high-q region (0.02–0.2 Å^−1^) was fitted using an ellipsoidal model (see Eq. S2 in the supplementary information). The equatorial radius varies with the D_2_O solvent contrast, decreasing from approximately 78 to 73 Å, while the polar radius was fixed at ∼13 Å. This trend may arise from changes in the pKₐ of d-C10 caused by isotope exchange. Furthermore, the q^−4^ scaling of I(q) in the linear region suggests the presence of large particles or aggregates within the sample.

Fig. 2B shows the scattering intensity profiles of 100 mM d-C10 systems measured at 0, 20, 40, 60, 80, and 100% D₂O solvent compositions. Although 100% D₂O provides low contrast for d-C10, measurable scattering is still observed, likely due to the presence of residual hydrogenated C10, as the sample is not fully deuterated. Similar to C10, d-C10 exhibits the coexistence of small micelles and larger aggregates. The high-q region (0.01–0.3 Å^−1^) was fitted using an ellipsoidal model (see Eq. S2 in the supplementary information). The polar radius (b-axis) was fixed at ∼13 Å during fitting, a value that corresponds closely to the extended molecular length of a single decanoate chain, consistent with a monolayer packing arrangement. The equatorial radius of d-C10 decreases monotonically from ∼164 Å at 0% D_2_O to ∼68 Å at 100% D_2_O (Table S1). Since varying the H₂O/D₂O ratio changes which components are visible to the neutron beam, this systematic trend suggests that the aggregates possess internal compositional heterogeneity: at 0% D₂O, where d-C10 dominates the scattering signal, the full spatial extent of the deuterated surfactant within the aggregate is probed, whereas at 100% D₂O, d-C10 is nearly contrast-matched and the residual scattering arises primarily from hydrogenated impurities.

The linear region of both C10 and d-C10 scattering profiles is proportional to q^−4^, which is characteristic of the presence of large droplets or aggregates (Fig. 2). These observations are in agreement with previous findings by Berg et al. (Berg et al., 2022), who reported the formation of C10 aggregates of varying sizes at pH 6.5, supporting the coexistence of multiple aggregated structures observed here. Cryo-TEM was employed to characterize the optically turbid solution. The images revealed heterogeneous vesicular structures with diameters ranging from 50 to 80 nm (Fig. S4-A), which were predominantly stacked on the carbon support film. These vesicles, being substantially larger than the SANS q-range can resolve, contribute to the q^−4^ scattering observed at low q.

At pH 8.5, C10 and d-C10 form small micelles in 100% D_2_O and 0% D_2_O contrast. The scattering intensity profiles are fitted to the sphere model (described in supplementary information, Eq. S2) with a radius of around 15 Å. Micelles of comparable size at this pH have been reported previously (Namani and Walde, 2005; Berg et al., 2022).

Moreover, the aggregation number, N_agg_, of the C10 aggregates was determined by dividing the aggregate volumes obtained from the fitted model parameters by the molecular volume of C10. The scattering profiles at pH 8.5 (Fig. S1 in the supplementary information) indicate an aggregation number of 37 molecules, in agreement with the previously reported value (Huang and Verrall, 1997). However, the reported literature value was obtained using apparent molar volume and adiabatic compressibility measurements, with pH-adjusted water (pH 9.5) employed as the solvent. In contrast, the aggregation number for the pH 6.5 system is significantly higher, reaching 4248 molecules, consistent with the formation of much larger ellipsoidal aggregates than the micelles observed at pH 8.5. The hundred-fold difference in N_agg_ between pH 8.5 and pH 6.5 reflects the fundamental transition from small charged micelles to large, partly neutralized aggregates, which is the central pH-dependent behavior that the rest of the paper will explore in multicomponent systems.

Overall, the scattering profiles indicate that the pH 6.5 systems form larger and more complex colloidal structures, including large droplets, vesicles, and ellipsoidal micelles. However, larger particles were not observed in the Cryo-TEM images, likely due to the limited grid thickness (∼100 nm). In contrast, the pH 8.5 systems predominantly exhibit small spherical micelles. The strong pH dependence of the self-assembly behavior of C10, resulting in substantial morphological changes, has also been observed for other amphiphilic surfactants, as reported by Patel et al. *(*Patel et al., *2021**)*. The following sections examine how these baseline aggregate structures are modified by the presence of intestinal fluid components and a therapeutic peptide.

### 3.3 C10 in fasted-state simulated intestinal fluid

**Fig. 3:**
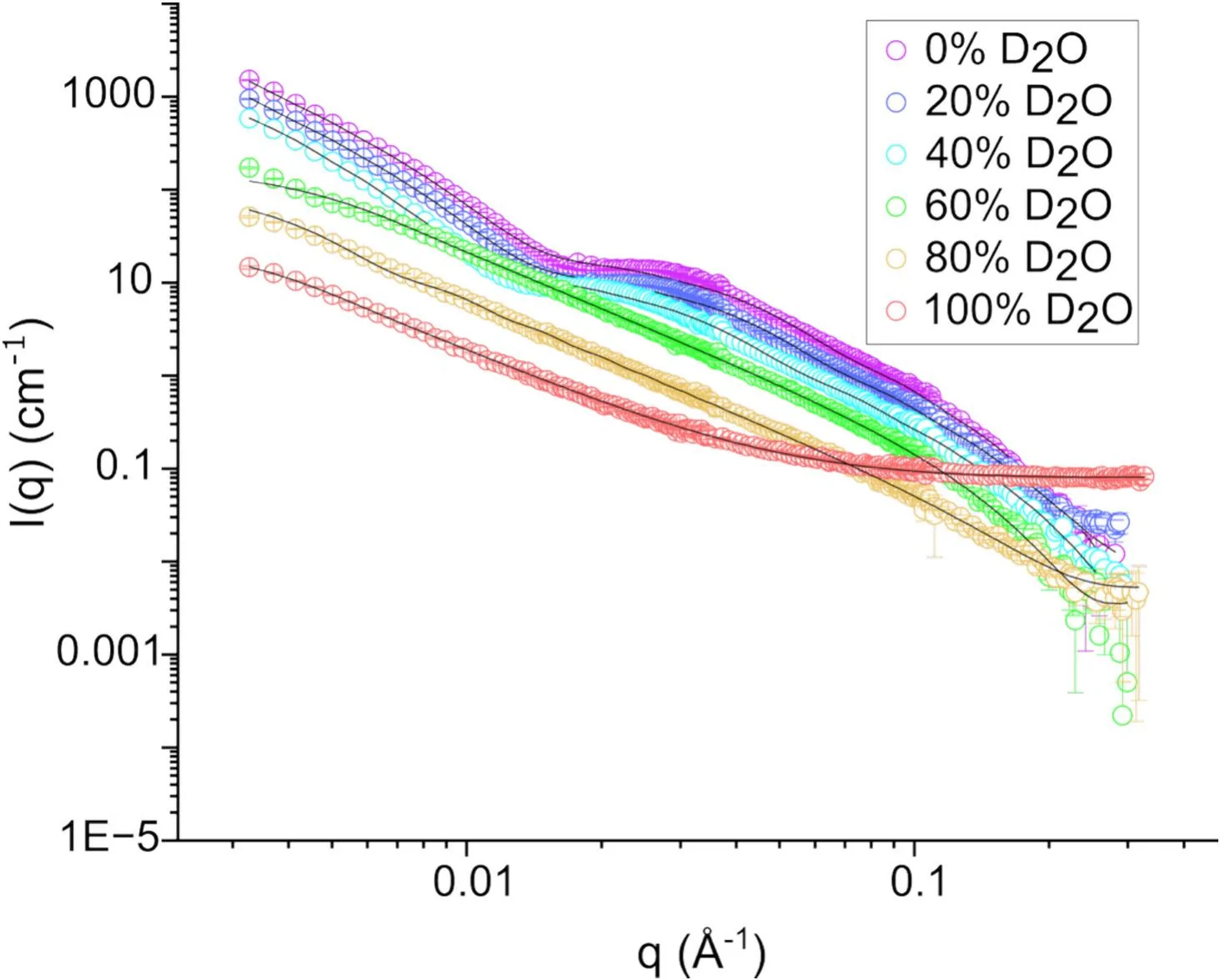
Small-angle neutron scattering (SANS) profiles and corresponding model fits for 100 mM d-C10 in fasted-state intestinal fluid at different D_2_O solvent contrasts. At high contrast (0–40% D_2_O), the scattering data are well described by an ellipsoidal model, whereas at low solvent contrast (60–100% D_2_O), the data are better represented by a cylindrical model.

Fig. 3 illustrates the scattering profiles of fasted-state intestinal fluid (FaSSIF) and *d*-C10 at different D₂O solvent contrasts. SANS in the q-range accessed here is primarily sensitive to the bilayer thickness and the lateral (in-plane) dimensions of the aggregates but cannot uniquely distinguish flat discs from closed vesicles without clear shell oscillations. The disc descriptors below should therefore be understood as referring to bilayer aggregates whose global topology is best determined by direct imaging. At pH 6.5, measurable scattering is detected across all solvent contrasts, indicating that the aggregates contain components with a range of scattering length densities. At high contrasts (0–40% D_2_O), the scattering profiles are best described by a two-ellipsoid model (see supplementary information, Eq. S2), indicating the coexistence of two distinct populations. Large ellipsoidal aggregates exhibit semi-axes of a = 1600–1946 Å (equatorial) and b = 220–280 Å (polar), whereas smaller ellipsoidal micelles have semi-axes of a = 66–85 Å and b = 11–12 Å.

In contrast, in low contrasts (60–100% D_2_O), the scattering data show no evidence of this coexistence. Instead, the profiles are well fitted using a cylindrical model (see supplementary information, Eq. S7 and S8). The fitted parameters reveal contrast-dependent radii between ∼300 and ∼ 600 Å and a cylinder length of approximately 20 Å, consistent with a bilayer morphology.

The bilayer thickness of approximately 20 Å obtained from the cylindrical fits at 60–100% D₂O (Table S2) is consistent with a bilayer arrangement of the aggregated molecules (Table S1). A thickness of 20 Å is consistent with an interdigitated C10 bilayer or a mixed bilayer of C10 with sodium taurocholate, which has a similar molecular length (13–17 Å), but is markedly smaller than the typical bilayer thickness of phospholipid systems (around 35–40 Å for DOPC). This suggests that the FaSSIF phospholipid component contributes less to the bilayer structure than C10 and the bile salt. The two-ellipsoid model is required at high contrast (0–40% D_2_O), where d-C10 dominates the scattering, while the cylindrical model applies at low contrast (60–100% D_2_O), where the FaSSIF components become visible. This contrast-dependent change in the apparent aggregate structure indicates that different components are highlighted at different solvent compositions, which is the expected signature of a co-assembled system in which C10 and the bile salt/lipid components are incorporated into a common aggregate. However, Cryo-TEM analysis of 100 mM C10 in FaSSIF at pH 6.5 revealed the presence of vesicles with diameters ranging from 50 to 200 nm (Fig. S4—B).

Riethorst et al. reported that FaSSIF exhibits a heterogeneous micellar population, with small ellipsoidal micelles (10–20 nm) and large ellipsoidal aggregates (20–50 nm) occurring in comparable proportions (Riethorst et al., 2016). Khoshakhlagh et al. (2015) demonstrated that FaSSIF, as a multicomponent system, can form vesicles and bicelles (Khoshakhlagh et al., 2015). In the present study, however, no clear distinction could be made between disk-like structures and large vesicles in mixtures of d-C10 and FaSSIF. Instead, the system displays pronounced structural heterogeneity, with the coexistence of multiple aggregate morphologies, including bilayer structures as well as large and ellipsoidal micelles. The structural heterogeneity observed here is markedly greater than that of either d-C10 alone (Section 3.2) or FaSSIF alone (Berg et al., 2021; Conrad et al., 2009), suggesting that co-assembly between C10 and the bile salt/phospholipid components generates aggregate morphologies that neither component produces independently.

At pH 8.5, the scattering profile of d-C10 in the presence of FaSSIF at 0% D₂O suggests the formation of small spherical micelles (Fig. S1). No measurable scattering was observed at 100% D₂O contrast, indicating that FaSSIF does not significantly contribute to the micellar structures under these conditions. The scattering profile was fitted with a sphere model (supplementary information, Eq. S2) with a radius of the micelle of ∼16 Å.

At pH 6.5, the large ellipsoidal aggregates formed at 0% D_2_O contrast contain more than 10^7^ molecules, whereas the bilayer aggregates observed at 100% D_2_O contrast exhibit an aggregation number N_agg_ of approximately 11,000 molecules, assuming a disc-like geometry for the calculation. The aggregation number was calculated from the aggregate volumes derived from the fitted model parameters and the average molecular volume of sodium taurocholate and lipids, assuming complete contrast matching of other components at 100% D_2_O.

At pH 8.5, the presence of FaSSIF also enhances aggregation, increasing N_agg_ to 54 molecules relative to the corresponding d-C10-only system.

Overall, d-C10 mixed with FaSSIF forms large, multicomponent colloidal structures at pH 6.5, whereas d-C10 alone forms large droplets, vesicles, and smaller ellipsoidal micelles. At pH 8.5, the aggregate morphology remains unchanged by the presence of FaSSIF; however, the aggregation number increases, indicating enhanced association without a change in aggregate shape.

### 3.4 C10 in fed-state simulated intestinal fluid

**Fig. 4:**
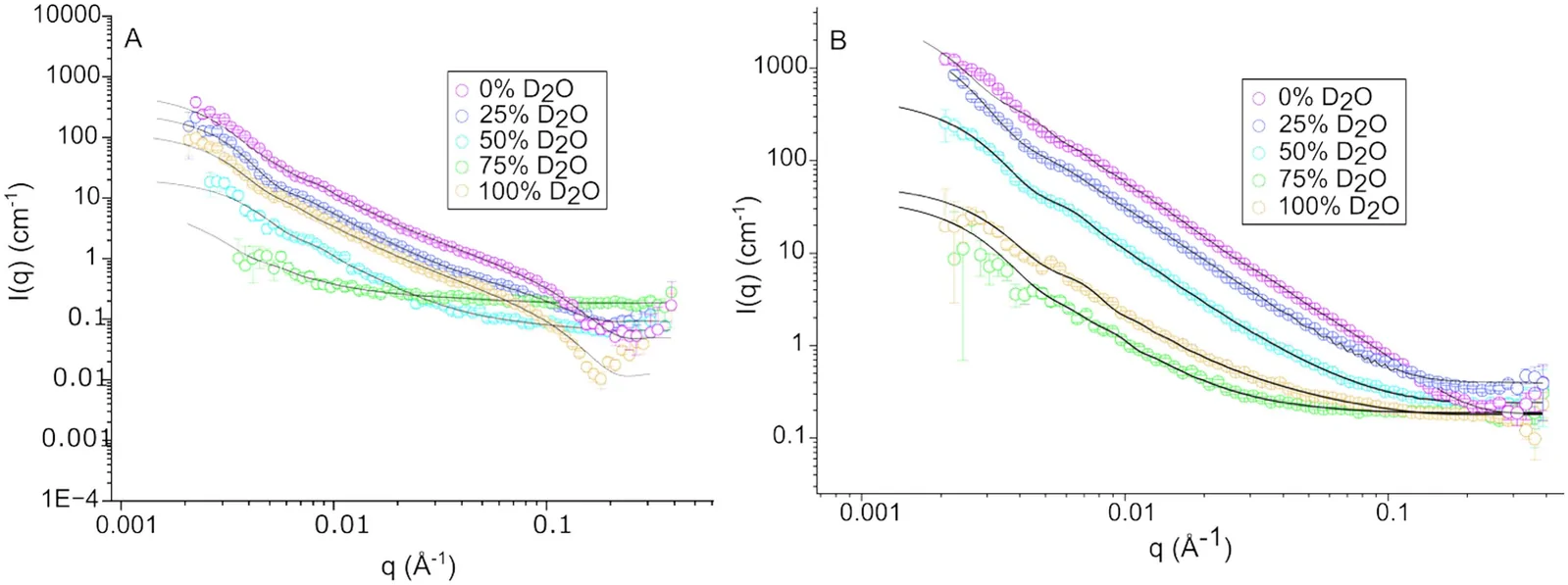
Small-angle neutron scattering (SANS) profiles and corresponding model fits for 50 mM (A) and 100 mM (B) d-C10 in fed-state intestinal fluid at pH 6.5 at different D_2_O solvent contrasts. The data were fitted using a bilayer model with coexisting small micelles.

The scattering profiles for fed-state intestinal fluid (FeSSIF) and d-C10 at concentrations of 50 mM and 100 mM, investigated at different solvent ratios, are presented in Fig. 4A and Fig. 4B, respectively. The SANS data for all samples were analyzed using models that accounted for bilayer coexisting with small micelles (Table 2).

In SANS measurements, the presence of vesicles with a well-defined bilayer shell structure typically results in characteristic oscillations in the scattering intensity in the low-q region. On this basis, the samples were assumed to consist predominantly of bilayers or discs with a thickness range of ∼20 Å to ∼30 Å (in different contrast, see supplementary information Table S3) and a minimum radius of approximately 500 Å, accompanied by an intensity-weighted fraction (typically less than 0.01) of small micelles with a radius in the range of ∼20–40 Å. The predominance of bilayer structures in the FeSSIF system, in contrast to the mixed ellipsoidal and bilayer structures observed with FaSSIF (Section 3.3), is consistent with the higher phospholipid content of FeSSIF favoring low-curvature bilayer geometries.

The 50 mM and 100 mM d-C10 systems exhibit similar aggregate morphologies, with bilayer morphologies dominating at both concentrations. The absolute scattering intensity and the fitted bilayer volume fractions increase with C10 concentration (supplementary information Table S3), indicating that more surfactant is incorporated into the bilayer aggregates at higher concentration, but without inducing new structural features. This suggests that the aggregate architecture is governed primarily by the FeSSIF components, with C10 contributing additional material to the existing bilayer framework. This interpretation is consistent with the findings of Riethorst et al. (Berg et al., 2021), who showed that human intestinal fluid under fed-state conditions contains taurocholic acid and lecithin as the dominant colloidal species, forming bile salt-phospholipid mixed micelles and lipid droplets. Since FeSSIF is designed to replicate this colloidal environment, the persistence of FeSSIF-dominated aggregate structures even at elevated C10 concentrations is not unexpected. In addition, Cryo-TEM images of both 50 and 100 mM C10 in FeSSIF revealed vesicular aggregates with diameters ranging from 10 to 50 nm, predominantly in the 40–50 nm range (supplementary information Fig. S4 C—D). However, the absence of a Guinier regime in the SANS data prevented a clear distinction between disc-like structures and vesicles. Consequently, the aggregation numbers reported for the disc/bilayer structures throughout this study should be regarded as minimum values.

Moreover, at pH 6.5, the aggregation numbers show a strong dependence on the contrast conditions. At 0% D₂O, where only d-C10 contributes to the scattering, an aggregation number (N_agg_) of approximately 185,000 is obtained, reflecting the number of d-C10 molecules within the bilayer, calculated assuming a disc-like structure.

This value is significantly lower than that observed when d-C10 forms large ellipsoidal aggregates, as discussed in Section 3.3. This difference likely reflects the change in aggregate morphology: the flat disc geometry accommodates fewer molecules per aggregate than the large globular ellipsoids formed in the FaSSIF system, even though the total amount of aggregated material may be comparable. In contrast, at 100% D₂O, the scattering is dominated by FeSSIF components, yielding an aggregation number of approximately 19,000, which corresponds to the number of FeSSIF molecules associated with the aggregates. This value is higher than that observed in systems containing FaSSIF, consistent with the higher concentrations of sodium taurocholate and lipids present under fed-state conditions.

**Fig. 5:**
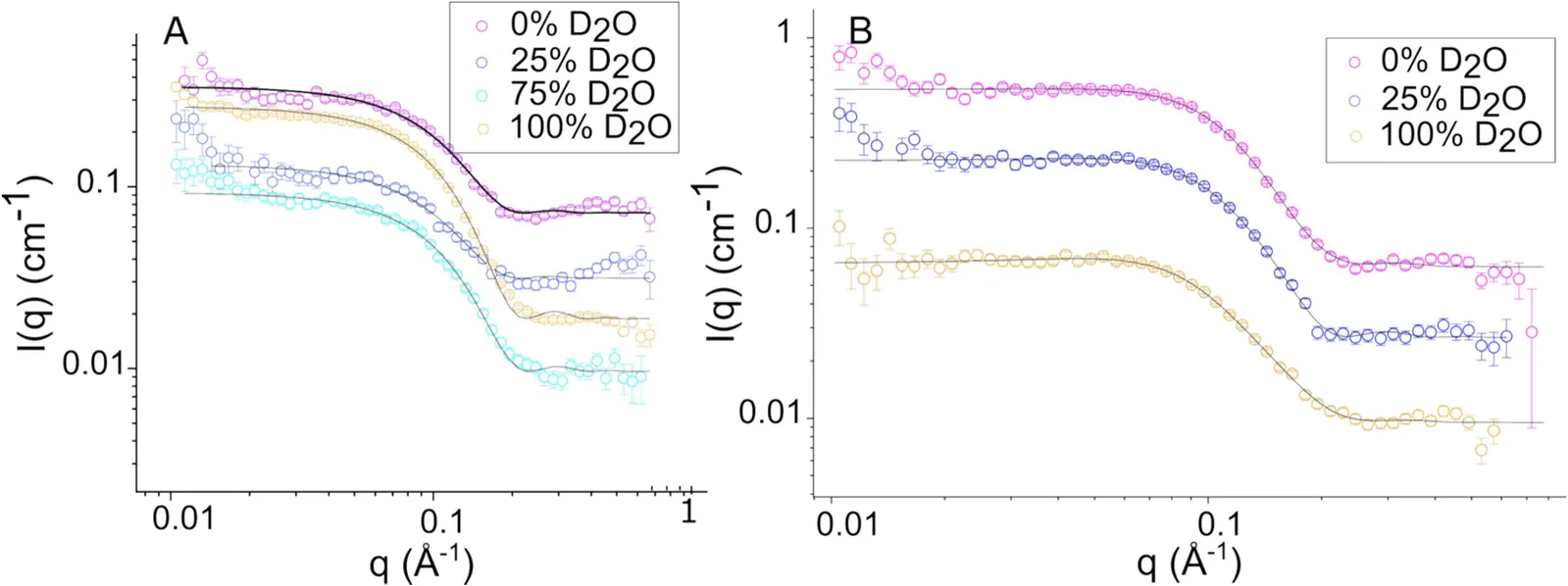
Small-angle neutron scattering (SANS) profiles and corresponding model fits for 50 mM (A) and 100 mM (B) d-C10 in fed-state intestinal fluid at pH 8.5 at different D_2_O solvent contrasts. All the datasets were described by the sphere model with or without considering the structure factor.

At the higher pH of 8.5, the scattering profiles indicate weaker interaction between the FeSSIF components and d-C10 molecules (Fig. 5A and B). Notably, even at this higher pH, the scattering profiles remain comparable to those obtained for 50 mM and 100 mM d-C10 in both conditions. All datasets could be fitted using a spherical micelle model with an average radius of ∼20 Å, although clear structure-factor effects were present in the 100 mM d-C10 and FeSSIF samples (Fig. 5B). Overall, no pronounced differences were detected between the d-C10-only system and those containing FaSSIF or FeSSIF, apart from a slight increase in the micellar radius in the presence of FaSSIF and FeSSIF.

Furthermore, the aggregation number was determined for both pH conditions. At the higher pH, distinct aggregation numbers were again observed under different D₂O contrast conditions, with values of ∼97 and ∼ 38 for 0% and 100% D₂O, respectively. As before, the 0% D₂O contrast primarily reflects d-C10, while the 100% D₂O contrast highlights FeSSIF, despite the micellar radius remaining essentially unchanged. Notably, the aggregation number of d-C10 under these conditions is relatively high compared to systems containing only d-C10 or d-C10 in the presence of FaSSIF.

### 3.5 C10 with octreotide

To determine whether the therapeutic peptide (octreotide) co-assembles as an amphiphilic cosurfactant, partitions into the micellar interior, or preferentially interacts with the micellar interface, the self-assembly behavior of octreotide when combined with C10 was investigated.

**Fig. 6:**
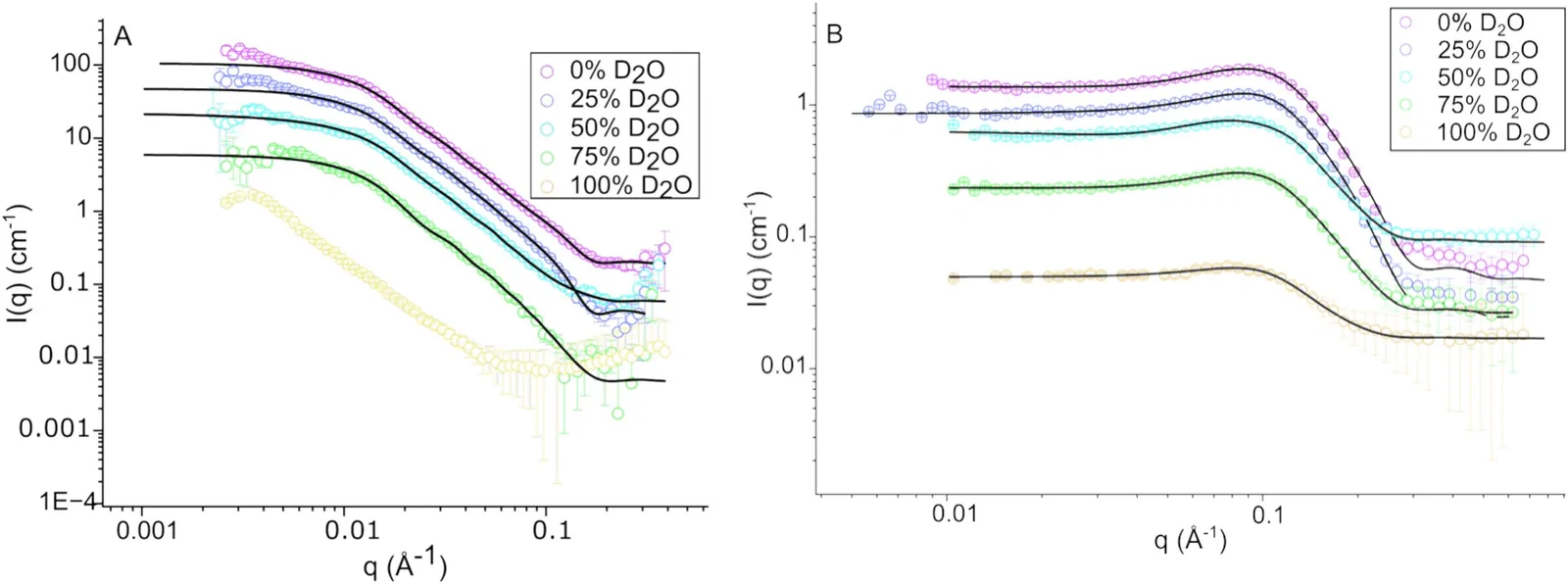
Small-angle neutron scattering (SANS) profiles and corresponding model fits for 300 mM d-C10 with the therapeutic peptide octreotide at pH 6.5 (A), and pH 8.5 (B), measured at different D_2_O solvent contrasts. At pH 6.5, the data are best described by a coexistence model comprising bilayers and micelles, whereas at pH 8.5, the scattering profiles are well captured by an ellipsoidal model. In all cases, the datasets were fitted using appropriate form-factor models, with structure-factor contributions included where necessary.

Fig. 6 illustrates the scattering for the therapeutic peptide octreotide and d-C10 systems. The effect of octreotide on C10 self-assembly is markedly more pronounced at pH 6.5 (Fig. 6A) than at pH 8.5 (Fig. 6B). The similarity of the scattering profiles across different D₂O solvent contrasts is consistent with the formation of mixed aggregates containing both octreotide and d-C10, as discussed further below.

At pH 6.5, the scattering data were fitted using a model comprising bilayer structures coexisting with small micelles. For 0–75% D₂O solvent contrast, the bilayer structures exhibited a minimum radius of approximately 168 Å, together with a small fraction (∼0.1, intensity-weighted fraction) of smaller micellar aggregates. The 100% D_2_O data exhibited similar scattering profiles; however, with d-C10 being contrast-matched, the scattering contribution arose predominantly from the small amount of octreotide. As a result, the data quality was insufficient for reliable model fitting.

Notably, as discussed in Section 3.2, d-C10 alone predominantly forms large droplets and ellipsoidal micelles, whereas in the presence of octreotide, it preferentially assembles into bilayer-based structures. This morphological transition, driven by the addition of only 2 mM octreotide relative to 300 mM C10, is an indication that octreotide behaves as a cosurfactant. The implications of this bilayer-forming tendency for drug loading and formulation stability remain to be investigated.

In addition, d-C10 in the presence of octreotide undergoes phase separation within 10 min. Consequently, it is possible that a fraction of the largest aggregates sedimented during the measurement, as the measurement time was around 30 min, such that these particles were not fully probed by the neutron beam. The reported aggregation numbers and volume fractions for this system should therefore be considered lower bounds.

The aggregation number was calculated only at 0% D₂O contrast and corresponds to approximately 18,000 d-C10 molecules assembled into a bilayer (calculated assuming a disc-like structure). This value is between the aggregation number observed in the FaSSIF (approximately 11,000 at 0% D_2_O) and FeSSIF systems (approximately 185,000 at 0% D_2_O). However, the pronounced morphological changes induced by octreotide and the rapid phase separation of the system suggest that specific hydrophobic and electrostatic interactions between the peptide and C10 also contribute to the enhanced aggregate growth (Sjögren et al., 2005).

All scattering profiles at pH 8.5 were fitted using an ellipsoidal model in which the polar axis (b) was approximately three to five times larger than the equatorial radius (a), corresponding to a prolate (rod-like) geometry (see Table S5 in the supplementary information). These fits indicate that d-C10 in the presence of octreotide forms prolate, rod-like micelles under these conditions. In contrast, C10 at the same concentration forms only spherical micelles at pH 8.5 (Fig. S2, supporting information). Similarly, d-C10 alone, as well as d-C10 in the presence of FaSSIF or FeSSIF, predominantly forms spherical micelles. The aggregation number of the rod-like d-C10–octreotide aggregates was approximately 116 molecules, which is higher than that observed for d-C10 micelles in the presence of FeSSIF (N_agg_ ∼ 97), and more than an order of magnitude higher than for d-C10 alone (N_agg_ ∼ 37).

The fitted semi-minor axis (a) of the rod-like d-C10–octreotide micelles at pH 8.5 is 13–14 Å across all D₂O contrasts (Table S5, supplementary information), identical to the value observed for pure C10 micelles and ellipsoids at both pH values. This invariance of the radial dimension is significant: it demonstrates that octreotide does not swell the micellar cross-section, and therefore does not insert into the hydrophobic core in a way that would increase the packing radius. Instead, octreotide promotes axial elongation, increasing the semi-major axis from approximately 15 Å (spherical micelles of C10 alone) to 48–70 Å without perturbing the radial packing. The fitted geometry (b > > a) corresponds to prolate, rod-like aggregates rather than oblate. This geometric constraint is consistent with octreotide acting as a cosurfactant that inserts at the aggregate interface and promotes uniaxial micellar growth, analogous to the role of cosurfactants in the sphere-to-rod transition observed in classical surfactant systems. If octreotide were instead adsorbing at the micellar surface without integrating into the aggregate interior, one would not expect such a pronounced and systematic elongation effect. Although the C10 concentration differs between the peptide-free (100 mM) and peptide-containing (300 mM) systems, the invariance of the micellar cross-sectional radius across contrasts together with the systematic elongation of the semi-major axis points to structural evidence for a peptide-specific effect.

**Fig. 7:**
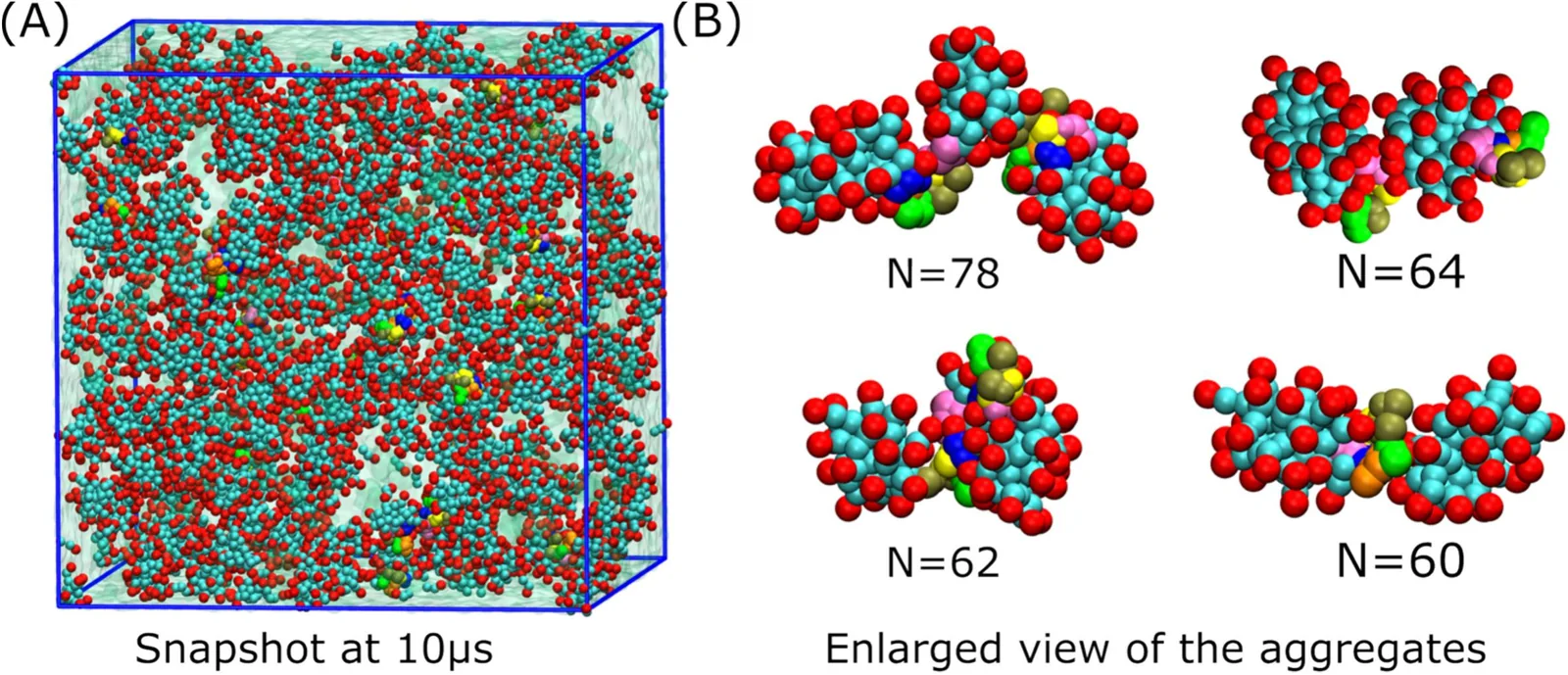
CG-MD simulation of C10–octreotide micelle formation. (A) Final snapshot of the simulation box containing C10 and octreotide molecules representing pH 8.5. (B) Representative enlarged views of the simulated micellar aggregates formed in the presence of octreotide. Color scheme: C10 tail beads, cyan; C10 headgroup beads, red; Phe, blue; N-terminal Phe, mauve; Lys, green; Thr, tan; Trp, orange; Cys, yellow. Water is shown as a light green surface. (For interpretation of the references to color in this figure legend, the reader is referred to the web version of this article.)

To further elucidate the formation of rod-like C10 aggregates in the presence of octreotide under pH 8.5 conditions, where all C10 molecules are fully ionized, we performed coarse-grained molecular dynamics (CG-MD) simulations. In the simulation, 300 mM C10 and 2 mM octreotide were randomly placed in a 40 × 40 × 40 nm^3^ simulation box and simulated for 10 μs. During the simulation, C10 molecules self-assembled into micelles, with octreotide molecules becoming incorporated into the aggregates. Interestingly, the simulations also revealed the formation of small rod-like aggregates (Fig. 7) with aggregation numbers ranging from 60 to 78. The semi-minor axis (a) of these micelles was ∼15 Å, while the semi-major axis (b) ranged from 40 to 50 Å, in good agreement with the SANS data.

Note that previous simulations of systems containing only C10 did not show the formation of such non-spherical micelles; instead, the predominant aggregates were spherical, with aggregation numbers of approximately 40 (Akter et al., 2026; Hossain et al., 2022). These findings suggest that, despite the limited simulation time and length scale, the simulations captured the early-stage formation of larger rod-like aggregates that experimentally reached aggregation numbers of approximately 116. Furthermore, the CG-MD simulations independently support the interpretation from the SANS data that octreotide acts as a co-surfactant within the C10–octreotide rod-like aggregates. An enlarged view of the simulated aggregates (Fig. 7B) shows that octreotide molecules localize at the interface of the C10 aggregates, with hydrophobic residues such as Phe and Trp partially inserted into the hydrocarbon tail region, while hydrophilic residues such as Lys and Thr remain oriented toward the aqueous phase. This interfacial positioning is consistent with co-surfactant behavior and corroborates the structural interpretation derived from the SANS contrast-variation data.

Overall, our results demonstrate that octreotide co-assembles with sodium caprate under both pH conditions and behaves in a manner consistent with an amphiphilic cosurfactant. This behavior contrasts with previous reports on structurally related peptides, such as lanreotide, which were shown to associate primarily with surfactant headgroups and to reduce the aggregation number (Brunzell et al., 2026). The different behavior of octreotide and lanreotide may be related to their physicochemical features (Bo et al., 2022). The hydrophobic and hydrophilic residues of octreotide are well distributed, with the Phe and Trp residues capable of interacting with the C10 hydrocarbon tails while the Lys and Thr residues are exposed to the water. It may therefore be included in the C10 interface and operate as a co-surfactant. In contrast, lanreotide does not have enough hydrophobic and hydrophilic domains to be aligned with the surfactant molecules and mostly interacts with the charged surface of the micelles instead of mixing into the interfacial layer. In the present system, however, the presence of octreotide in combination with sodium caprate induces pronounced changes in nanoparticle size and morphology and leads to a substantial increase in aggregation. These findings highlight the peptide-specific nature of surfactant–peptide interactions and their strong influence on self-assembly behavior.

### 3.6 Comparative structural analysis of C10 colloidal systems

Taken together, the results reveal a consistent pattern in the pH-dependent self-assembly of C10 across all environments studied. At pH 6.5, where a substantial fraction of C10 is in its unionized (protonated) form, the aggregates are uniformly large and structurally complex. Pure C10 forms ellipsoidal aggregates and vesicles coexisting with large droplets (N_agg_ ≈ 4200). The addition of FaSSIF drives the system toward even larger mixed aggregates with N_agg_ exceeding 10^7^ at 0% D₂O contrast and approximately 11,000 for the FaSSIF-visible bilayer component. FeSSIF pushes the morphology further toward bilayer/discs (N_agg_ ≈ 185,000 for d-C10 at 0% D₂O), and octreotide produces the discs/bilayer structures with the aggregation numbers (N_agg_ ≈ 18,000). The structural progression proceeds from small micelles that increase in length, to the coexistence of small micelles and large bilayer structures, and finally to predominantly large bilayer aggregates as amphiphilic co-components are added. The micelle-to-bilayer transition can be rationalized in terms of changes in spontaneous curvature. In the octreotide-containing systems, the insertion of octreotide as a cosurfactant at the C10 aggregate interface reduces the effective surface charge density, which alters the intermolecular packing and favors the formation of lower-curvature aggregates. The spontaneous curvature is reduced through co-assembly of C10 with the bile salt and phospholipid components, which have intrinsically lower spontaneous curvature than C10 alone. In both cases, the result is a shift from high-curvature micellar morphologies toward lower-curvature bilayer aggregates.

At pH 8.5, by contrast, all systems converge to small spherical micelles with radii of ∼15–20 Å and aggregation numbers in the range of 37–97, regardless of the presence of FaSSIF or FeSSIF. For the d-C10–octreotide system, prolate, rod-like aggregates with N_agg_ ≈ 116 are formed. The dominance of the ionized form at this pH produces a high surface charge density that favors small, high-curvature micelles and largely overrides the influence of co-components. This convergence has implications for the colloidal context in which C10 acts as a permeation enhancer. At pH values approaching those of the distal small intestine (pH 7–8), the C10 aggregates are small, morphologically simple, and largely insensitive to intestinal fluid composition, whereas at the lower pH values encountered in the proximal intestine and stomach, the aggregate landscape is far more heterogeneous. Whether the free monomer concentration is the dominant factor for permeation enhancement, or whether the aggregates themselves contribute to drug interaction and transport, has been the subject of active investigation. Berg et al. (Berg et al., 2022) reported that vesicular C10 outperformed micellar C10 in enhancing macromolecular absorption in rat intestine at low C10 concentrations, and Cunha et al. (Cunha et al., 2026) recently proposed that micelles and vesicles enhance mucosal permeation through distinct mechanisms: micelles disrupt the epithelial lipid membrane rapidly, while vesicles fluidize the membrane without compromising it and interact strongly with the mucus layer. Bardonnet, Sassene and colleagues (Bardonnet et al., 2026) developed a pH-modifier-based C10 tablet designed to favor the micellar form in the gastric environment, though the anticipated advantage over SNAC did not translate fully from acid-pretreated dogs to humans. The structural characterization presented here does not resolve which colloidal form is functionally optimal, but the quantitative aggregation numbers, dimensions, and contrast-variation-derived compositions provide a structural foundation on which these mechanistic observations can be interpreted. In particular, the sensitivity of C10 aggregate structure to intestinal fluid composition at pH 6.5, and the dramatic morphological reorganization induced by a therapeutic peptide, indicate that the functional picture cannot be understood without accounting for the specific colloidal context in which C10 operates in vivo.

## 4 Conclusions

This study characterized the self-assembly of sodium caprate (C10) under intestinally relevant conditions using contrast variation SANS and light scattering. Three principal findings emerge. First, the aggregate morphology at pH 6.5 is highly sensitive to the surrounding medium, progressing from ellipsoidal micelles, vesicles, and droplets (C10 alone) through mixed ellipsoidal and bilayer structures (with FaSSIF) to predominantly bilayer discs (with FeSSIF or octreotide), while at pH 8.5, all systems except C10-octreotide converge to small spherical micelles. Second, contrast variation analysis reveals that the FaSSIF and FeSSIF aggregates are co-assembled structures containing both C10 and bile salt/phospholipid components, rather than independent populations. Third, octreotide behaves as an amphiphilic cosurfactant under both pH conditions, as evidenced by the dramatic morphological changes it induces and the invariance of the micellar cross-sectional radius, which indicates that the peptide does not occupy substantial volume within the hydrophobic interior. These findings demonstrate that peptide-excipient interactions can fundamentally alter aggregate architecture and should be considered in the design of caprate-based formulations for oral peptide delivery. This is a particularly relevant question in light of recent in vivo comparisons showing that C10 underperforms SNAC as an enhancer for oral octreotide delivery in rats (Fredholt et al., 2026). Future work should address the relationship between the aggregate structures characterized here and the permeation-enhancing efficacy of C10, particularly the question of whether drug loading into the bilayer-like assemblies offers advantages over free monomer-based delivery.

## CRediT authorship contribution statement

**Shahina Akter:** Writing – review & editing, Writing – original draft, Visualization, Investigation, Formal analysis, Data curation. **L. Magnus Bergström:** Writing – review & editing, Supervision, Software, Resources, Project administration, Formal analysis, Data curation. **Per Hansson:** Writing – review & editing, Resources, Methodology, Investigation. **Joachim Kohlbrecher:** Writing – review & editing, Supervision, Software, Resources, Investigation. **James Doutch:** Writing – review & editing, Resources, Methodology, Investigation. **Shakhawath Hossain:** Writing – review & editing, Writing – original draft, Visualization, Supervision, Software, Methodology, Investigation, Formal analysis, Data curation. **Per Larsson:** Writing – review & editing, Supervision, Resources, Project administration, Methodology, Funding acquisition, Conceptualization.

## Funding

The authors acknowledge financial support from Swedish National Graduate School in Neutron Scattering (SwedNess), 10.13039/501100001729Swedish Foundation for Strategic Research (SSF) GSn15 – 0008. Also, financial support from 10.13039/501100001858VINNOVA (2019-00048) for the Swedish Drug Delivery Center (SweDeliver) is gratefully acknowledged.

## Declaration of competing interest

The authors declare that they have no known competing financial interests or personal relationships.

that could have appeared to influence the work reported in this paper.

## Data Availability

Data will be made available on request.
