# Supplementary material for: Self-assembly of sodium caprate in simulated intestinal fluids and with a therapeutic peptide: A small-angle neutron scattering study
Source: Int J Pharm X. 2026 Aug 16;12:100641. doi: 10.1016/j.ijpx.2026.100641 (PMC13521064; doi:10.1016/j.ijpx.2026.100641)
Supplement: Supplementary file 1 [file mmc1.docx]

**Self-Assembly of sodium caprate in simulated intestinal fluids and with a therapeutic peptide: A small-angle neutron scattering study**

Shahina Akter^1^, L. Magnus Bergström^2^, Per Hansson^2^, Joachim Kohlbrecher^3^, James Doutch^4^, Shakhawath Hossain^1^, Per Larsson^1^

^1^Department of Pharmacy, Uppsala University, 751 23 Uppsala, Sweden

^2^Department of Medicinal Chemistry, Uppsala University, Box 574, SE-751 23 Uppsala, Sweden

^3^PSI Center for Neutron and Muon Sciences, 5232 Villigen PSI, Switzerland

^4^ISIS Neutron and Muon Source, STFC, Rutherford Appleton Laboratory, Harwell Campus, Didcot OX11 0QX, Oxon, United Kingdom

Figure S1: Scattering profiles of 100 mM C10, d-C10, d-C10+ FaSSIF at pH 8.5 in different contrasts.

Figure S2: Scattering profile of 300 mM C10 at pH 8.1 in 100%-D_2_0

Figure S3: SLS and SANS profiles for 100 mM C10 (A), 100 mM C10 + FaSSIF (B), 50 mM C10 + FeSSIF (C), 100 mM C10 + FeSSIF (D), and 300 mM C10 + Octreotide (E).

Figure S4: Cryo-TEM images of sodium caprate (C10) at different compositions. (A) 100 mM C10 at pH 6.5 showing heterogeneous vesicles with diameters ranging from 50 to 80 nm, predominantly stacked on the carbon support film. (B) 100 mM C10 in FaSSIF at pH 6.5 showing vesicles with diameters ranging from 50 to 200 nm. (C) and (D) 50 mM C10 and 100 mM C10 in FeSSIF showing vesicles with diameters ranging from 10 to 50 nm, with the majority of vesicles in the 40–50 nm range.

Table S1: Fitting result for the systems of 100 mM C10 and d-C10 with the Ellipsoid model.

| **D_2_O solvent ratio** | **100 mM C10** | | **100 mM d-C10** | |
| --- | --- | --- | --- | --- |
|  | **a-axis (Å)** | **b-axis (Å)** | **a -axis (Å)** | **b -axis (Å)** |
| 0 |  |  | 164.060 ± 0.440 | 13 ± 0.343 |
| 20 |  |  | 122.450 ± 0.356 | 13 ± 0.444 |
| 40 |  |  | 94.692 ± 0.372 | 13 ± 0.461 |
| 60 | 78.272 ± 0.398 | 13 ± 609 | 80.874 ± 0.408 | 13 ± 0.545 |
| 80 | 74.280 ± 0.321 | 13 ± 0.347 | 68.178 ± 0.392 | 13 ± 0.560 |
| 100 | 72.943 ± 0.310 | 13 ± 0.220 | 67.528 ± 0.696 | 13 ± 0.666 |

Table S2: Fitting results for the system of 100 mM d-C10 with FaSSIF

| **D_2_O solvent ratio** | **Large Ellipsoid** | | **Small Ellipsoid** | | **Bilayer** | |
| --- | --- | --- | --- | --- | --- | --- |
|  | **a -axis (Å)** | **b -axis (Å)** | **a -axis (Å)** | **b -axis (Å)** | **Radius (Å)** | **Thickness(Å)** |
| 0 | 1946.80 ± 25.344 | 220.88 ± 0.381 | 66.755 ± 0.087 | 12.5 ± 0.150 |  |  |
| 20 | 1946.6 ± 24.761 | 232.84 ± 0.447 | 70.699 ± 0.090 | 11.756 ± 0.122 |  |  |
| 40 | 1600.5 ± 16.756 | 280.65 ± 0.641 | 85.118 ± 0.119 | 11.40 ± 0.140 |  |  |
| 60 |  |  |  |  | 300 ± 4.792 | 22.613 ± 0.166 |
| 80 |  |  |  |  | 600.43 ± 69.22 | 20.087 ± 0.395 |
| 100 |  |  |  |  | 394.9 ± 21.588 | 20 ± 1.043 |

| **D_2_O solvent ratio** | **Bilayer** | | | |
| --- | --- | --- | --- | --- |
|  | **Radius^X^ (Å)** | | **Half-thickness (Å)** | |
| **C10 Conc. (mM)** | **50** | **100** | **50** | **100** |
| 0 | 1010 ± 1 | 1286 ± 6 | 15 ± 1 | 10.49 ± 5.88 |
| 25 | 825 ± 18 | 810 ± 1 | 15 ± 1 | 15 ± 1 |
| 50 | 511.8 ± 1 | 653 ± 1 | 10 ± 1 | 11.37 ± 1 |
| 75 | 998.1 ± 1 | 923 ± 1 | 10 ± 1 | 15 ± 1 |
| 100 | 865.8 ± 1 | 729 ± 1 | 13.9 ± 1 | 12 ± 1 |

Table S3: Fitting results for the systems of 50 and 100 mM C10 with FeSSIF

^X^: Radius is bigger than the specified value in Table S3.

| **D_2_O solvent ratio** | **Bilayer** | |
| --- | --- | --- |
|  | **Radius ^X^ (Å)** | **Half-thickness (Å)** |
| 0 | 168.7 ± 1 | 35.6 ± 1 |
| 25 | 168.8 ± 1 | 37.2 ± 1 |
| 50 | 163.1 ± 1 | 25.4 ± 2 |
| 75 | 166.5 ± 2 | 14.8 ± 1.6 |
| 100 | Didn’t manage to fit | |

Table S4: Fitting results for the systems of 300 mM C10 with Octreotide at pH 6.5

^X^: Radius is bigger than the specified value in Table S4.

| **D_2_O solvent ratio** | **Rod-like (prolate) Micelles** | |
| --- | --- | --- |
|  | **b -axis (Å)** | **a-axis (Å)** |
| 0 | 57.9 ± 0.51 | 13.1 ± 0.07 |
| 25 | 48 ± 3.00 | 13.48 ± 0.68 |
| 50 | 70.1 ± 0.04 | 13.7 ± 2.74 |
| 75 | 58.6 ± 0.95 | 13.6 ± 0.15 |
| 100 | 53 ± 5.7 | 14.2 ± 0.90 |

Table S5: Fitting results for the systems of 300 mM C10 with Octreotide at pH 8.5

**Least Squares Fitting Model:**

The differential scattering cross section as a function of scattering vector *q* for a sample of non-interacting monodisperse micelles with concentration *n* (number of micelles per unit volume) can be written as follows [1]:

$\frac{d\sigma\left( q \right)}{d\Omega}=n{\Delta\rho}^{2}V^{2}P\left( q \right)\left[ 1+\beta\left( q \right)\left( S\left( q \right)-1 \right) \right]$	(S1)

where Δ*ρ* is the difference in scattering length density between self-assembled aggregates and the medium and $P\left( q \right)$ is the form factor considering the shape of the aggregates. Interparticle correlations were accounted for by the structure factor S(*q*). The Hayter–Penfold rescaled mean spherical approximation (RMSA) was employed to describe the structure factor within the decoupling approximation with parameter *β*(*q*).

1. **Spheroidal and spherical micelles**

For micelles modeled as monodisperse and homogeneous ellipsoids of revolution (spheroids) with equatorial radius *a* and polar radius *b*, the orientationally averaged form factor is [1]

$P\left( q \right)=\left\langle F^{2}\left( q \right) \right\rangle=\int_{0}^{\pi/2} F{(q,\phi)}^{2}\sin\phi d\phi$	(S2)

and

$\beta\left( q \right)=\frac{\left\langle F\left( q \right) \right\rangle^{2}}{\left\langle F^{2}\left( q \right) \right\rangle}$	(S3)

The amplitude of the form factor is defined as

$F_{sph}\left( qr \right)=\frac{3(\sin qr-qr\cos qr)}{{(qr)}^{3}}$	(S4)

where

$r=\sqrt{a^{2}{sin}^{2}\phi+b^{2}{cos}^{2}\phi}$	(S5)

In the special case of monodisperse spheres

$P\left( q \right)=F_{sph}^{2}\left( qr \right)$	(S6)

2. **Bilayer discs modelled as flat cylinders**

The form factor of the randomly oriented cylindrical particles can be expressed as [1]

$P\left( q \right)=F^{2}\left( q \right)=\int_{0}^{\pi/2} F^{2}\left( q,\phi\right)\sin\phi d\phi$	(S7)

where

$F\left( q,\phi\right)=2\left( \Delta\rho\right)\frac{\sin\left( \frac{1}{2}qL\cos\phi\right)}{\frac{1}{2}qL\cos\phi}\frac{B_{1}(qR\sin\phi)}{qR\sin\phi}$	(S8)

Here, *φ* represents the angle between the cylinder’s axis and the vector *q*. The cylinder has a volume *V* = *πR*²*L*, where *L* is the flat cylinder thickness and *R* is the radius. Δ*ρ* (the contrast) denotes the difference in scattering length density between the particle and the surrounding solvent. *B*₁ refers to the first-order Bessel function.

3. **Bilayers and coexistence with micelles**

In the coexistence region, the model is composed of form factors for micelles and bilayer disks written as [1]

$P\left( q \right)=x_{mic}P_{mic}+x_{disk}P_{bil}P_{disk}$	(S9)

where *x_mic_* and *x_disk_* are the relative intensity-weighted fractions of micelles and bilayer disks, respectively, with the constraint *x_mic_* + *x_disk_* = 1. [2, 3].

The form factor of a homogeneous bilayer cross-section with half-bilayer thickness $\xi$ equals

$P_{bil}\left( q \right)=\left( \frac{sinq\xi}{q\xi} \right)^{2}$	(S10)

The form factor of infinitely thin disk with disk radius *R_d_* equals

$P_{disk}=\frac{2[1-B_{1}(2qR_{d})/qR_{d}]}{{(qR_{d})}^{2}}$	(S11)

and *B*_1_(*x*) is the Bessel function of first order.

The agreement between the experimental data and the model was quantified using the reduced chi-squared parameter, defined as

$\chi^{2}=\frac{1}{N-M}\sum_{i=1}^{N} \left( \frac{I_{exp}\left( q_{i} \right)-I_{mod}\left( q_{i} \right)}{\sigma_{i}} \right)^{2}$	(S12)

where *I_exp_*(*q_i_*) and *I_mod_*(*q_i_*) are the experimental and model intensities, respectively, at a scattering vector modulus *q_i_*, and $\sigma$*_i_* is the statistical uncertainty on the data points, *N* is the total number of data points, and *M* is the number of parameters optimized in the model fit.

**References:**

1.	Pedersen, J.S., *Analysis of small-angle scattering data from colloids and polymer solutions: modeling and least-squares fitting.* Advances in Colloid and Interface Science, 1997. **70**: p. 171–210.

2.	Bergström, L.M., et al., *Spontaneous Transformations between Surfactant Bilayers of Different Topologies Observed in Mixtures of Sodium Octyl Sulfate and Hexadecyltrimethylammonium Bromide.* Langmuir, 2014. **30**(14): p. 3928–3938.

3.	Bergström, M. and J.S. Pedersen, *A Small-Angle Neutron Scattering Study of Surfactant Aggregates Formed in Aqueous Mixtures of Sodium Dodecyl Sulfate and Didodecyldimethylammonium Bromide.* The Journal of Physical Chemistry B, 2000. **104**(17): p. 4155–4163.
